# Molecular epidemiology of *Streptococcus pneumoniae* isolated from pediatric community-acquired pneumonia in pre-conjugate vaccine era in Western China

**DOI:** 10.1186/s12941-020-00410-x

**Published:** 2021-01-06

**Authors:** Zhuoxin Liang, Jinjian Fu, Ling Li, Rongsong Yi, Shaolin Xu, Jichang Chen, Xiaohua Ye, Eric McGrath

**Affiliations:** 1 Department of Pediatric Intensive Care Unit, Department of Prevention and Health Care, Department of Pediatric, Department of Laboratory, Department of Neonatology, Liuzhou Maternity and Child Health Care Hospital, Liuzhou, China; 2 Department of Social Medicine and Health Management, Xiangya School of Public Health, Central South University, Changsha, China; 3 Laboratory of Molecular Epidemiology, School of Public Health, Guangdong Pharmaceutical University, 283# Jianghai Dadao, Haizhu District, 510310 Guangzhou, China; 4 Children’s Hospital of Michigan, Detroit, MI USA; 5 Wayne State University School of Medicine, Detroit, MI USA

**Keywords:** Community-acquired pneumonia, *Streptococcus pneumoniae*, Children, Vaccine, Molecular characteristics

## Abstract

**Background:**

*Streptococcus pneumoniae* (*S. pneumoniae*) is one of the most common pathogens which can cause morbidity and mortality in pediatric infections worldwide. This study aimed to describe the phenotypic and molecular characteristics of community-acquired pneumonia (CAP)-causing *S. pneumoniae* recovered from children in Western China.

**Methods:**

We retrospectively enrolled pediatric patients younger than 5 years diagnosed with CAP. All 419 *S. pneumoniae* isolates were tested for antibiotic susceptibility, serotypes, virulence genes, resistance genes, and sequence types. The potential relationships between molecular characteristics were tested by correspondence analysis.

**Results:**

Most of *S. pneumoniae* isolates were resistant to erythromycin, tetracycline, clindamycin and trimethoprim-sulfamethoxazole, with 93.8% isolates classified as multidrug resistant. The dominant STs were ST271 (30.8%) and ST320 (12.2%), while the prevailing serotypes were 19F (46.8%), 6B (11.5%), 23F (9.5%) and 19A (9.3%). The coverage rates of PCV-7 and PCV-13 were 73.03% and 86.16%, while the coverage rates of PCV13 among children aged < 1 year and 1–2 years were high in 93.18% and 93.62%. We also observed that CC271 expressed more of *mef* (A/E), *lytA*, *rlrA* and *sipA* than non-CC271 isolates. Moreover, there were strong corresponding relationships between molecular characteristics.

**Conclusions:**

The high coverage rate of PCV13 suggests the necessity of introducing the PCV13 vaccine in Western China. Our findings underscore the value of monitoring multiple molecular characteristics to provide new guidance for developing future pneumococcal vaccines.

##  Introduction

Community-acquired pneumonia (CAP) is a common childhood disease around the world. The overall percentage of CAP and the causative pathogens varied geographically, especially in developing countries. According to the World Health Organization (WHO), there are more than 900,000 deaths per year in children under than 5 years who have suffered from CAP [1]. *Streptococcus pneumoniae* (*S. pneumoniae*) is one of the most common causes of bacterial CAP in children and was of great concern in pediatric CAP, accounting for 2.2–50.9% of cases younger than 5 years [2].

Pneumococcal conjugate vaccines (PCVs) targeting 7, 10, or 13 of more than 90 serotypes of *S. pneumoniae* have been successively introduced to reduce the pediatric pneumococcal disease burden around the world [3]. PCV7 vaccination was associated with a 19% and 33.1% reduction in the rate of CAP in children aged < 5 years and aged < 2 years, respectively, in the UK. But for unvaccinated children, there was no significant reduction in the incidence of CAP [4]. After introduction of PCV13 into the national immunization program in the USA, there was a 21% reduction in hospital admissions for all-cause CAP in children aged < 2 years, suggesting a positive impact of PCV immunization on CAP in children [5], especially for those caused by unique PCV13 serotypes of pneumococcal isolates which was not covered within PCV7, which were further decreased across each age group [3].

The highest prevalence of CAP was recorded in Chinese children aged < 6 months around the developing countries (37.88%) [6] and *S. pneumoniae* was responsible for 5.2% of CAP in children aged < 5 years [7]. Although PCVs can reduce the burden of pneumococcal diseases, the PCV7 was not included as part of the national immunization schedule due to its high price in China. The coverage of PCV7, − 10, and − 13 was 62.6%, 64.8% and 79.6%, respectively before the licensing of PCV7 [8]. The coverage of PCV7, − 10, and − 13 was changed into 58.6%, 59.4% and 85.1%, respectively in Shanghai [9]. The PCV7 was imported into and licensed in China in 2008, but immunization rates were less than 10% in published studies. The PCV7 vaccine was removed from the market in 2015 and PCV13 was available in some big cities, but in Western China, such as Liuzhou, this vaccine has not been introduced yet.

Despite a well-developed knowledge of serotypes and antibiotic susceptibility of *S. pneumoniae* reported in China, little has been known on the potential relationship between STs, serotypes and molecular characteristics such as pilus genes. Therefore, we conducted this study of *S. pneumoniae* causing CAP in children aged < 5 years to characterize the antimicrobial susceptibility, serotypes, ST profiles, virulence genes and pilus gene of *S. pneumoniae* isolates, so as to provide implication for the formulation of multivalent pneumococcal vaccines.

## Materials and methods

### Study area and population

This retrospective study was conducted from January 2015 and January 2017 in two tertiary hospitals of Liuzhou. Almost 80% of the children infections have been treated in these two hospitals in Liuzhou city. A suspicion of CAP was based on at least one of the following symptoms: new onset of systemic infection such as chills, pain, sweat, or temperature > 38℃, and at least one of the acute lower respiratory tract infection symptoms (such as cough, chest pain, dyspnea, respiratory secretions, and abnormal auscultation). The suspicion CAP was confirmed for the chest radiograph or a computerized tomographic scan of the chest [10]. The eligibility criteria for enrollment into the study were as follows: (1) diagnosed as CAP (based on above criteria); (2) aged less than 60 months; (3) sputum, blood or alveolar lavage fluid specimens cultured and isolated *S. pneumoniae*; and (4) all children not vaccinating against *S. pneumoniae*.

*S. pneumoniae* isolates were classified as multidrug-resistant (MDR) if they were resistant to 3 or more classes of antibiotics. PCVs coverage were defined as coverage of identified serotypes of all the isolates in this study.

### Specimen culture, identification and antimicrobial susceptibility testing

Specimens were collected by the physicians or nurses and delivered to the clinical microbiology laboratory within 30 minutes. The specimens were cultured onto Columbia Agar with 5% sheep blood plates and placed in 35 °C, 5% CO_2_ incubated for 24 h to 48 h. Blood cultures were incubated using the BacT/Alert 3D system (bioMerieux). The positive pathogens were selected and incubated onto Columbia Agar with 5% sheep blood plates. *S. pneumoniae* was identified by the VITEK 2 compact automatic microbial analysis system (Biomérieux, Marcyl’ Etoile, France). The antimicrobial susceptibility testing was conducted according to the previous study (penicillin, vancomycin, erythromycin, levofloxacin, tetracycline, trimethoprim-sulfamethoxazole, chloramphenicol, and cefotaximea) [11]. E-test method was added to test clindamycin and linezolid according to the previous study [12].

### Serotyping

Multiplex polymerase chain reaction (m-PCR) methods was used to identified all pneumococcal isolate serotypes. The primers and reaction conditions were used as described in previous studies [13, 14]. *CpsA* gene found in all known pneumococcal serotypes was used as the positive control.

### Antimicrobial resistance genes and virulence genes detecting

The macrolide-resistant genes (*erm*(A), *erm*(B) and *mef*(A/E)), tetracycline resistance genes (*tet*(K), *tet*(L) and *tet*(O)) were amplified by PCR methods, and the primers and PCR conditions were used as previously described [15, 16]. Virulence genes (*ply*, *pasA*, *lytA*, and *pspA*) and pilus genes (*rlrA* for PI-1 and *sipA* for PI-2) were detected as previous studies described [15].

### Multilocus sequence typing

Multilocus sequence typing (MLST) of the seven housekeeping genes (*aroE, gdh, gki, recP, spi, xpt*, and *ddl*) was conducted using primers and protocols as previously described [17]. Allelic profiles and STs were assigned by querying the pneumococcal MLST database (https://pubmlst.org/spneumoniae).

### Statistical analysis

Categorical variables were compared using Pearson’s chi-squared (χ^2^) test or Fisher exact test. Correspondence analysis was used to determine the internal relations between the serotypes and STs, and between STs and pilus genes. A two-sided *P*-value < 0.05 was considered as being of statistical significance. All statistical analyses were conducted using Stata version 14.0 (Stata Corp LP, College Station, Texas, USA).

### Ethics statement

The study was approved by the Ethics Committee of Liuzhou Maternity and Child Healthcare Hospital, and it was performed in accordance with the approved guidelines as described previously [12]. The informed consents were signed by guardian before the enrollment.

## Results

### Demographic and clinical characteristics of study participants

A total of 419 children with *S. pneumoniae* CAP were included. A total of 259 boys (61.8%) and 160 (38.2%) girls were suffered from *S. pnuemoniae* CAP. The ages of study participants ranged from 0 to 5 years, with average age 1.39 ± 1.19 years. The frequency distribution of age groups was 184 (43.9%) aged < 1 year, 132 (31.5%) aged 1–2 years, and 103 (24.6%) aged > 2 years.

### STs, serotypes and PCVs coverage

**Fig. 1:**
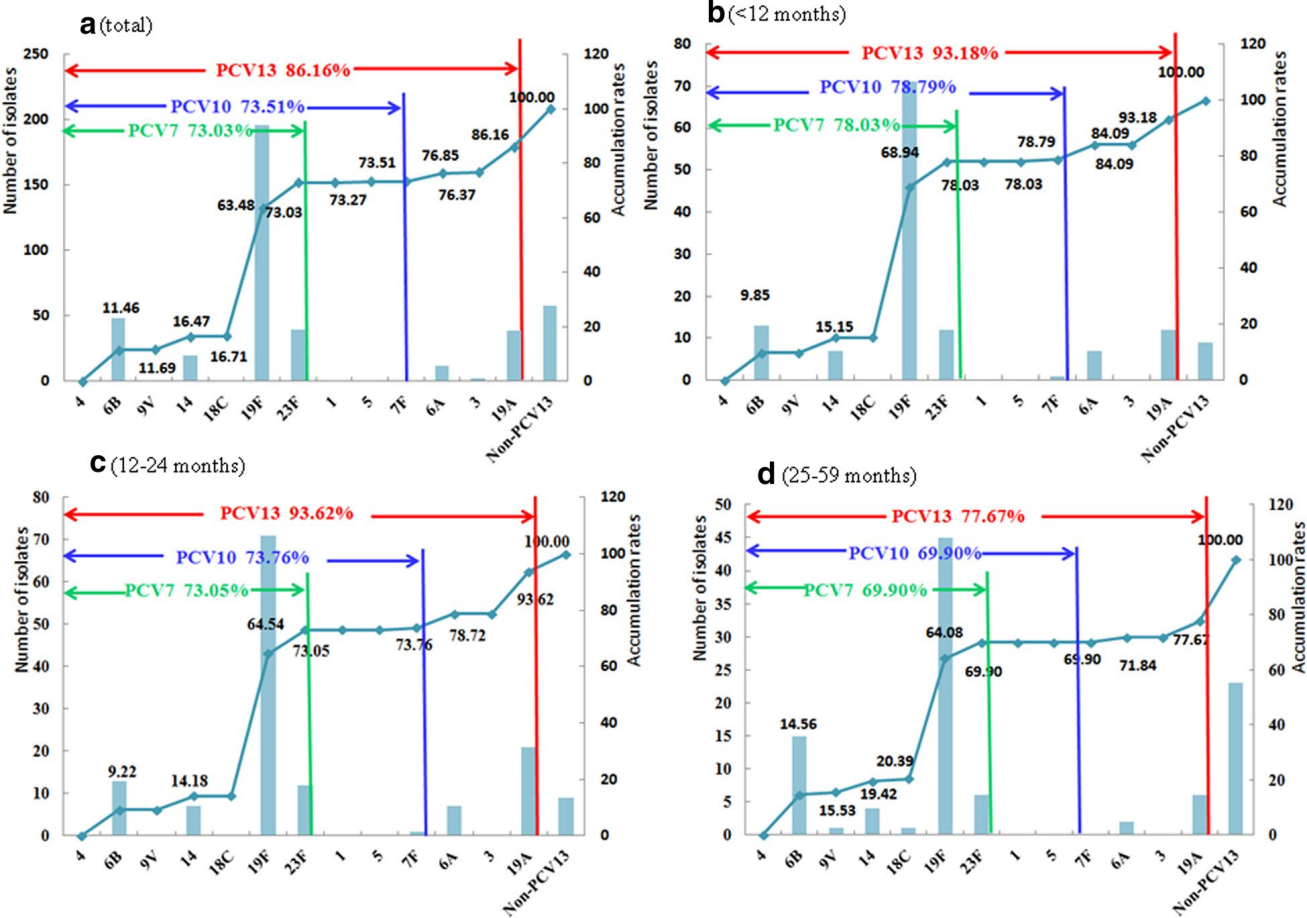
Serotypes distribution and PCVs coverage among CAP isolates sorted by ages. **a** total children < 60 months of age; **b** children < 12 months of age; **c** children 12–24 months of age; **d** children 25–59 months of age

There were 79 sequence types (STs) detected in this study. The dominant STs were ST271 (30.8%) and ST320 (12.2%). The most important clonal complex (CC) was CC271 (182 isolates, 43.4%). There were 15 serotypes detected in this study, and the prevailing serotypes were 19F (46.8%), 6B (11.5%), 23F (9.5%), and 19A (9.3%). The coverage rates of PCV-7, PCV-10 and PCV-13 were 73.03%, 73.51% and 86.16%, respectively (Fig. 1). The coverage rates of PCV7 among CAP children aged < 1 year, 1–2 years and > 2 years were 78.03%, 73.05% and 69.90%, respectively, while the coverage rates of PCV13 among 3 groups were 93.18%, 93.62% and 77.67%, respectively (Fig. 1).

### Antimicrobial resistance and virulence genes

There were 94.3%, 87.8%, 94.7% and 81.1% isolates resistant to erythromycin, tetracycline, clindamycin and trimethoprim-sulfamethoxazole, respectively (Table 1). The resistance rate of tetracycline, trimethoprim-sulfamethoxazole, clindamycin and cefotaxime in the CC271 group was significantly higher than that in the non-CC271 group (*P <* 0.05, Table 1).

**Table 1:** Antimicrobial susceptibility of *S. pneumoniae* isolated from children with CAP

Antibiotic	CC271 *n* (%) (n = 182)		Non-CC271 *n (*%) (n = 237)		Total *n* (%) (n = 419)		χ^2^	*P*
	R	S	R	S	R	S		
Penicillin Parenteral^a^	5 (2.7)	177 (97.3)	1 (0.4)	236 (99.6)	6 (1.5)	413 (98.5)	2.47	0.116
Vancomycin	0 (0.0)	182 (100.0)	0 (0.0)	237 (100.0)	0 (0.0)	419 (100.0)	–	–
Erythromycin	175 (96.2)	7 (3.8)	220 (92.8)	17 (7.2)	395 (94.3)	24 (5.7)	2.11	0.146
Levofloxacin	2 (1.1)	180 (98.9)	2 (0.8)	235 (99.2)	415 (99.0)	4 (1.0)	0.00	1.000
Tetracycline	169 (92.9)	13 (7.1)	200 (84.4)	37 (15.6)	368 (87.8)	51 (12.2)	7.03	*0.008*
Trimethoprim-sulfamethoxazole	167 (91.8)	15 (8.2)	202 (85.2)	35 (14.8)	369 (88.1)	50 (11.9)	4.17	*0.041*
Linezolid	15 (8.2)	167 (91.8)	20 (8.4)	217 (91.6)	35 (8.4)	384 (91.6)	0.01	0.942
Clindamycin	177 (97.3)	5 (2.7)	220 (92.8)	17 (7.2)	397 (94.7)	22 (5.3)	4.05	*0.044*
Chloramphenicol	7 (3.8)	175 (96.2)	14 (5.9)	223 (94.1)	21 (5.0)	398 (95.0)	0.92	0.338
Cefotaxime^a^	81 (44.5)	101 (55.5)	39 (16.5)	198 (83.5)	120 (28.6)	299 (71.4)	39.63	* < 0.001*

Italic values indicate significance of *P* value (*P* < 0.05)

CAP: community-acquired pneumonia

^a^2016 CLSI breakpoints were considered for non-meningitis

There was 93.8% *S. pneumoniae* classified as MDR, and the most common MDR profile was erythromycin-tetracycline-clindamycin-trimethoprim-sulfamethoxazole and erythromycin-tetracycline-clindamycin-trimethoprim-sulfamethoxazole-cefotaxime (Table 2). Penicillin retained high levels of susceptibility in tested isolates. Among all MDR isolates, the coverage rates of PCV7, PCV10, and PCV13 were 73.8%, 74.3% and 87.3%, respectively.

**Table 2:** Multidrug resistance patterns of *S. pneumoniae* isolated from children with CAP

Resistance patterns	No.(%)	Related serotypes (No.)
P-E-TE-DA-SXT-CTX	4 (0.9)	19F (3), 23F (1)
P-E-LEV-TE-DA-SXT-CTX	1 (0.2)	19F (1)
P-E-DA-SXT	1 (0.2)	19F (1)
E-TE-DA-SXT-LZD-CL	1 (0.2)	6B (1)
E-TE-DA-SXT-LZD	17 (4.1)	6B (1), 19A (4), 19F (10), 23F (2)
E-DA-SXT-LZD	10 (2.4)	6B (2), 15B/C (2), 15A (1), 19A (1), 19F (2), 23F (2)
E-DA-SXT	2 (0.4)	19F (2)
E-SXT-LZD	1 (0.2)	19F (1)
E-LEV-TE-DA-SXT-CTX	2 (0.4)	3 (1), 19F (1)
E-LEV-TE-DA	1 (0.2)	UT (1)
E-TE-DA-SXT-CL-CTX	7 (1.7)	6A (1), 19F (6)
E-TE-DA-SXT-CL	11 (2.6)	6A (2), 6B (5),14 (1), 19F (1), 23F (1), 35B (1)
E-TE-DA-CL	1 (0.2)	7F (1)
E-TE-SXT-CTX	2 (0.4)	19F (2)
E-TE-DA-CTX	1 (0.2)	19F (1)
TE-DA-CTX	1 (0.2)	19A (1)
E-TE-DA-SXT-CTX	101 (24.1)	6A (2),6B (2),14 (1),15B/C (4),18C (1),19A (10), 19F (72),23F (6), UT (3)
E-TE-DA-SXT	184 (24.1)	5 (1),6A (4), 6B (29),9V (1), 14 (7),15A (5), 15B/C (10), 19A (20), 19F (68), 23A (2), 23F (23), 34 (3), 35B (3),UT (8)
E-TE-SXT-DA	4 (0.9)	19F (2), 23A (1), 23F (1)
E-TE-SXT	2 (0.4)	6B (1), 19F (1)
E-DA-SXT	17 (4.1)	6B (4), 14 (3),19A (1),19F (7), 23F (1),35B (1)
E-DA-SXT	22 (5.3)	3 (1), 6A (2), 6B (1), 14 (5),15A (1),19A (2),19F (7),23A (3)
Total number of isolates	393 (93.8)	

P: penicillin G; E: erythromycin; TE: tetracycline; DA: clindamycin; SXT: trimethoprim-sulfamethoxazole; CTX: cefotaxime; LEV: levofloxacin; LZD: linezolid; CL: chloramphenicol; UT: untypeable;

There were 91.9%, 57.0% and 95.9% isolates carrying *erm*(B), *erm*f(A/E) and *tet*(M) genes, respectively (Table 3). There were more CC271 isolates carried *mef*(A/E) than non-CC271 isolates (*P* < 0.001), while there were more non-CC271 isolates carried *tet*(L) gene than CC271 isolates (*P* = 0.021). There were 403 (96.2%) isolates carried *lytA* gene, the carriage rate of *lytA* gene in the CC271 group was higher than that in the non-CC271 group (*P* < 0.001). The carriage rate of *rlrA* in the CC271 group was higher than that in the non-CC271 group (*P* < 0.001), and similar differences between CC271 and non-CC271 isolates were found for the *sipA* gene.

**Table 3:** Antibiotic resistance genes and virulence genes of *S. pneumoniae* isolated from children with CAP

Variables	CC271 n (%) (n = 182)		Non-CC271 n (%) (n = 237)		Total n (%) (n = 419)		χ^2^	*P*
	Positive	Negative	Positive	Negative	Positive	Negative		
*erm*(B)	168 (92.3)	14 (7.7)	217 (91.6)	20 (8.4)	385 (91.9)	34 (8.1)	0.08	0.781
*mef* (A/E)	156 (85.7)	26 (14.3)	83 (35.0)	154 (65.0)	239 (57.0)	180 (43.0)	107.96	< *0.001*
*tet* (M)	178 (97.8)	4 (2.2)	224 (94.9)	13 (5.1)	402 (95.9)	17 (4.1)	1.61	0.205
*tet* (K)	0 (0.0)	182 (100.0)	3 (1.3)	234 (98.7)	3 (0.7)	416 (99.3)	0.26	0.180
*tet* (L)	0 (0.0)	182 (100.0)	7 (3.0)	230 (97.0)	7 (1.7)	412 (98.3)	5.46	*0.021*
*tet* (O)	3 (1.6)	179 (98.4)	7 (3.0)	230 (97.0)	10 (2.4)	409 (97.6)	0.29	0.586
*ply*	171 (94.0)	11 (6.0)	211 (89.0)	26 (11.0)	382 (91.2)	37 (8.8)	3.10	0.078
*lytA*	182 (100.0)	0 (0.0)	221 (93.2)	16 (6.8)	403 (96.2)	16 (3.8)	12.77	< *0.001*
*psaA*	8 (4.4)	174 (95.6)	7 (3.0)	230 (97.0)	15 (3.6)	404 (96.4)	0.62	0.431
*pspA*	123 (67.6)	59 (32.4)	150 (63.3)	87 (36.7)	273 (65.2)	146 (34.8)	0.84	0.361
*rlrA*	91 (50.0)	91 (50.0)	48 (20.3)	189 (79.7)	139 (33.2)	280 (66.8)	41.09	< *0.001*
*sipA*	154 (84.6)	28 (15.4)	32 (13.5)	205 (86.5)	186 (44.4)	233 (55.6)	210.89	< *0.001*

Italic values indicate significance of *P* value (*P* < 0.05)

CAP: community-acquired pneumonia

### Relationship between STs, serotypes, and pilus genes

**Fig. 2:**
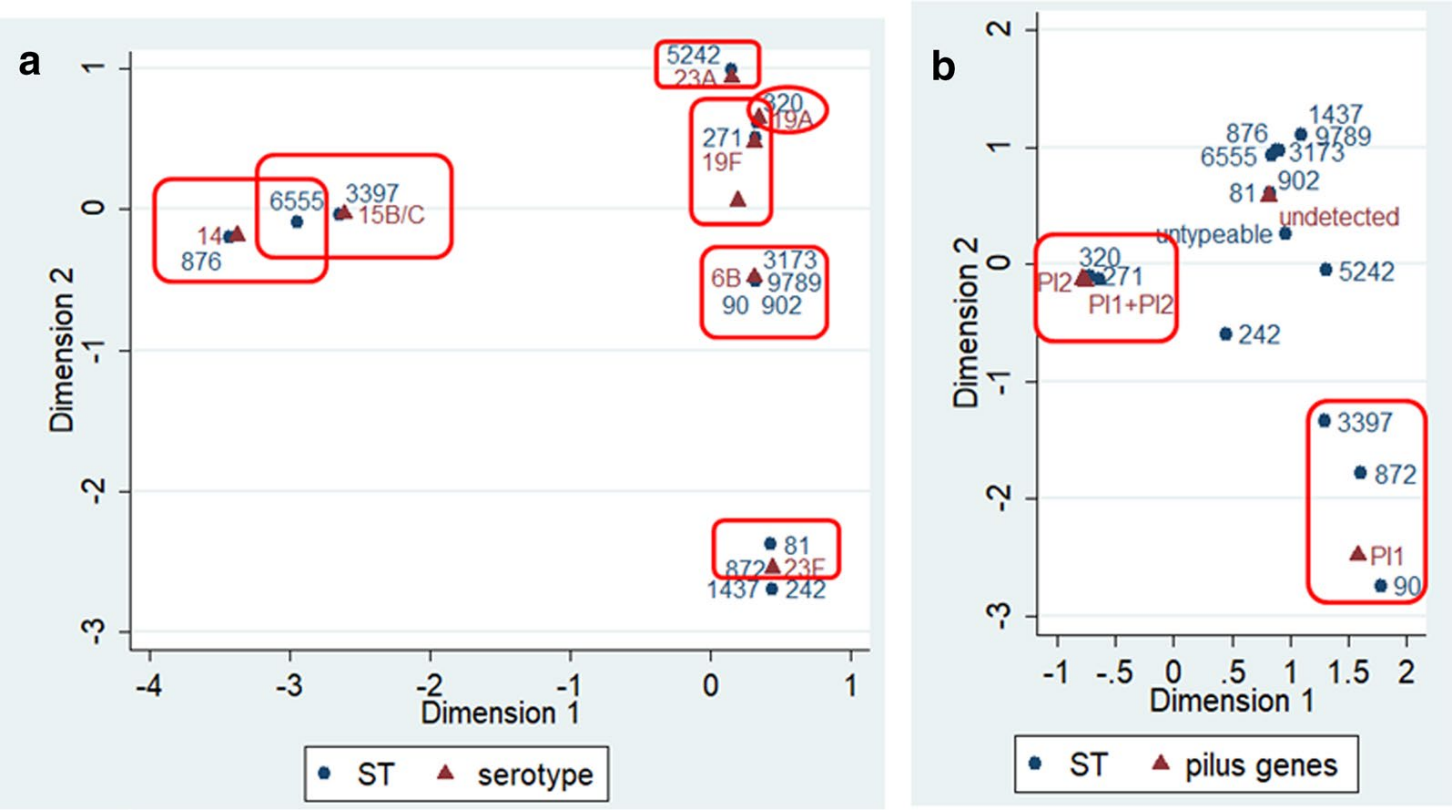
Correspondence analysis for the relationship between serotypes and STs (**a**), and pilus genes and STs (**b**)

The correspondence analysis indicated that there was a significant corresponding relationship between serotypes and STs (*χ*^*2*^ = 255.59, *P* < 0.001; Fig. 2). For example, serotype 19A was associated with ST320, serotype 23F was associated with ST81, serotype 6B was associated with ST90/ST902/ST3173/ST9789, serotype 15B/C was associated with ST3397, and serotype 19F was associated with ST271 and ST320. Additionally, we also revealed a significant corresponding relationship between STs and PIs (such as ST90/ST872/ST3397 and PI-1, ST320/271 and PI-1 + PI-2, and ST320/271 and PI-2; *χ*^*2*^ = 127.27, *P* < 0.001; Fig. 2).

## Discussion

This study demonstrated that most of the *S. pneumoniae* isolated from CAP were resistant to erythromycin, tetracycline, clindamycin, and trimethoprim-sulfamethoxazole, and the MDR rate was as high as 93.8%. The prevalent STs were ST271 and ST320, whereas the predominant serotypes were 19F, 6B, 23F, and 19A. The total coverage of PCV13 was 86.16%. The positive carriage rates of *mef*(A/E), *lytA*, *rlrA* and *sipA* were higher in CC271 isolates than that in non-CC271 isolates. The most interesting findings were that we found strong corresponding relationships between serotypes and STs, and between STs and PIs.

In this study, the predominant STs were ST271, ST320, and ST81, which were all MDR. The predominant CC was CC271, accounting for 41.4% of all the isolates. It was reported that the CC271 clone belonged to the known Taiwan19F^14^, which has been described as the major factor to spread MDR isolates internationally, including China [11, 18]. Our data showed that over 90% isolates, including the CC271 clone were MDR. Most of these isolates were resistant to erythromycin, tetracycline and trimethoprim-sulfamethoxazole, and over 90% isolates carried both *erm*(B) and *tet*(M) genes. Previous study revealed that *Tn6002* was a result of the insertion of the *erm*(B) and *Tn916* was a result of the insertion of the macrolide efflux genetic element *mef*(E), and both of which carried *tet*(M) and were identical to the CC271 clone [19]. It was interesting that CC271 clone carried higher *mef*(A/E) rate than the non-CC271 clone, which was consistent with other studies[11, 20]. Reports from both Asian Network for Surveillance of Resistant Pathogens (ANSORP) and China revealed that the *S. pneumoniae* isolates had high resistance rate to the macrolides and tetracycline antibiotics [12, 21, 22]. The combination of the above results suggest that both genetic background and antibiotic selective pressure had contributed to the spread of the CC271 clone.

It is believed that vaccination is not only a crucial way to reduce the burden of pneumococcal diseases in children, but also has a broad herd immunity effect in the whole population [23]. A vast number of epidemiological studies revealed that the incidence of pneumococcal diseases which were vaccine-covered serotypes decreased significantly after large-scale vaccination of PCV7 in the USA and Europe, especially the serotypes of 19F, 6B, 23F and 14 [24, 25]. In this study, the potential coverage rates of PCV7 and PCV10 against CAP isolates were 73.0% and 73.5%, respectively. This indicates that the PCV10 would not add much extra benefit than PCV7 if it is introduced into Western China, because the extra serotypes included in PCV10 (serotype 1, 5 and 7F) were rarely detected in our study. However, PCV13 would potentially provide 13% of additional coverage of identified serotypes among children aged < 5 years and even 20% of additional coverage among children aged 1–2 years because serotype 19A was highly prevalent in this age group.

It was previously reported that the Taiwan 19F^− 14^ clone related to pneumococcal serotype 19A and 19F, which were spread extensively across China prior to the introduction of PCV7 and contributed to the prevalence of MDR *S. pneumoniae* isolates in China [26, 27].This positive association between the increase in serotypes 19A and 19F and the high epidemic of MDR CC271 isolates was also confirmed in other Asian countries such as Japan and South Korea before and after the introduction of PCV7 [28–31]. In developed countries such as the USA and Europe, the long-term effect of broad vaccination with PCV7 vaccine led to the increase of non-vaccine serotypes (serotype replacement), especially MDR-related serotype 19A [32]. Based on these findings, the vaccine immunization and protection effect could not entirely explain the emergence and dissemination of MDR-related serotypes of 19A and 19F, which related to the homogeneous genetic background of CC271 in Asian countries [31]. Evidence from multicenter surveillance in China demonstrated that 84.7% of the isolates of 19A and 19F belonged to CC271 [33], suggesting that in the area of where PCV7 vaccination rate of less than 10%, the vaccine implication pressure could not entirely explain these phenomenon, and other risk factors (such as antibiotic use and younger age) may promote the dissemination of these isolates among children suffering from CAP [34]. Our data suggest that the PCV13 coverage rate was 87.3% for all the MDR isolates, indicating that the PCV13 may have a potential benefit to reduce the spread of MDR isolates.

Recent evidence has revealed a potential association between pneumococcal serotypes and STs. Serotype 19F/ST271 prevails in China, Czech Republic, Iceland, and South Africa [11, 35–37], serotype 19A/ST320 was prevalent in Asia and worldwide [32, 38], serotype 14/ST876 was prevalent in China [39], and serotype 23F/ST81was prevalent in Taiwan [40]. A good consistency between pneumococcal serotypes and STs was revealed in this study using the correspondence analysis. The consistency was also reported in previous studies, mostly in China [11, 32, 34, 39].

Pilus islet has capable of assisting *S. pneumoniae* collapsing mucosa, triggering mucosal inflammation and adherence to host tissue, which make *S. pneumoniae* more virulent. Previous study revealed that the pilus genes were associated with certain complex clonal of *S. pneumoniae*. For example, the *rlrA* and *sipA* which encoded the PI-1 and PI-2 pilus islet have previously been described as positive among the Taiwan 19F-14 clone (CC271) [41]. Another study also found that the PCV7 non-vaccine serotype (19A) of *S. pneumoniae* also carried the pilus islet genes [42]. Consistent with above findings, our data demonstrated that CC271 has a great consistency with PI-1 and PI-2 pilus islets and carrying *mef*(A/E) gene. In addition, we found that certain pilus genes have corresponding relationships with certain STs, such as ST90/ST872/ST3397 and PI-1, ST320/271 and PI-1 + PI-2, and ST320/271 and PI-2. These findings indicate that pilus islets help the high virulence isolates such as CC271 spread among children suffered from CAP in Western China, and imply that the introduction of PCV13 would decrease the disease burden of CAP in this area.

This study has some limitations. First, this surveillance of CAP data was conducted only in two hospitals, which may limit the generalizability of our results. Results from this study need to be confirmed in future multiprovince or multinational surveillance studies. Second, some serotypes have not been identified, and further studies are needed to identify them in the future. However, the significant consistency between STs and serotypes and between STs and PIs of *S. pneumoniae* gives us a new insight into the spread of dominant complex clones among the children suffering from CAP. The time span of the sample collection was between January 2015 and January 2017; that is after the PCV7 was pulled off the Chinese market in 2015, but before PCV13 was introduced into Liuzhou, so this study can reflect the epidemiology of *S. pneumoniae* isolated from pediatric community-acquired pneumonia in pre-conjugate vaccine era in Western China.

In conclusion, this is the first study to focus on pediatric CAP*-*causing *S. pneumoniae* in Western China. Our findings indicate that *S. pneumoniae* CC271 isolates carry more resistance genes and virulence genes than non-CC271. Interestingly, there are strong corresponding relationships between serotypes and STs and between STs and PIs, which may provide new guidance for developing future pneumococcal vaccines. In addition, the high coverage rate of PCV13 suggests the necessity of introducing the PCV13 vaccine in Western China, which may reduce disease burden of CAP in this population.

## Data Availability

We declare that the data supporting the conclusions of this article are available from the corresponding author on reasonable request.

## Abbreviations

*S. Pneumoniae*: *Streptococcus pneumoniae*
CAP: Community-acquired pneumonia
WHO: World Health Organization
PCVs: Pneumococcal conjugate vaccines
MDR: Multidrug-resistant
m-PCR: Multiplex polymerase chain reaction
MLST: Multilocus sequence typing
STs: Sequence types
CC: Clonal complex
ANSORP: Asian network for surveillance of resistant pathogens
